# Involvement of Nrf2-HO-1/JNK-Erk Signaling Pathways in Aconitine-Induced Developmental Toxicity, Oxidative Stress, and ROS-Mitochondrial Apoptosis in Zebrafish Embryos

**DOI:** 10.3389/fphar.2021.642480

**Published:** 2021-04-21

**Authors:** Qing Xia, Shuo Gao, Samuel Rajendran Rapael Gnanamuthu, Kaiyan Zhuang, Zhenzhen Song, Yun Zhang, Xue Wang, Pengfei Tu, Jianheng Li, Kechun Liu

**Affiliations:** ^1^ Biology Institute, Qilu University of Technology (Shandong Academy of Sciences), Jinan, China; ^2^ Engineering Research Center of Zebrafish Models for Human Diseases and Drug Screening of Shandong Province, Jinan, China; ^3^ Shandong Provincial Engineering Laboratory for Biological Testing Technology, Jinan, China; ^4^ School of Pharmacy, Hebei University, Baoding, China; ^5^ State Key Laboratory of Natural and Biomimetic Drugs, School of Pharmaceutical Sciences, Peking University, Beijing, China

**Keywords:** aconitine, developmental toxicity, oxidative stress, mitochondrial apoptosis, zebrafish

## Abstract

Aconitine (AC), one of the bioactive diterpenoid alkaloids extracted from *Aconitum* plants, is widely used in traditional herbal medicine to treat various diseases. Emerging evidence indicates that AC has attracted great interest for its wide cardiotoxicity and neurotoxicity. However, the toxic effects of AC on embryonic development and its underlying mechanisms remain unclear. Here, a developmental toxicity assay of AC was performed on zebrafish embryos from 4 to 96 h post fertilization (hpf), and its underlying mechanisms were discussed. AC exposure impaired the cardiac, liver, and neurodevelopment. Especially, a high dose of AC (7.27 and 8.23 μM) exposure resulted in malformations at 72 and 96 hpf, including reduced body length, curved body shape, pericardial edema, yolk retention, swim bladder and brain developmental deficiency, and degeneration of dopaminergic neurons. High-concentration AC exposure caused a deficient cardiovascular system with cardiac dysfunctions, increased heart rates at 72 and 96 hpf, and reduced locomotor behavior at 120 hpf. AC treatment significantly increased the ROS level and triggered cell apoptosis in the heart and brain regions of embryos at 96 hpf in 7.27 and 8.23 μM AC treatment zebrafish. Oxidative stress was confirmed by reduced levels of T-SOD activity associated with accumulation of lipid peroxidation in larvae. The expression levels of oxidative stress-related genes (*Nrf2*, *HO-1*, *Cat*, and *Sod-1*) *Erk1/2* and *Bcl-2* were significantly downregulated at 96 hpf. The expression pattern of JNK and mitochondrial apoptosis-related genes (*Bad*, *Bax*, *Cyto C*, *Casp-9*, and *Casp-3*) was significantly upregulated. Taken together, all these parameters collectively provide the first evidence of AC-induced developmental toxicity in zebrafish embryo/larvae through ROS-medicated mitochondrial apoptosis involving Nrf2/HO-1 and JNK/Erk pathways.

## Introduction

*Aconitum* plants are commonly known as monkshood and widely distributed all over the world. It has been used as a traditional medicine to treat shock caused by acute myocardial infarction, coronary heart disease, and angina pectoris in China (Singhuber et al., 2009; Zhou et al., 2015; Chan, 2016; Liu et al., 2017). Aconitine (AC) is an extremely toxic compound isolated from *Aconitum*. The toxicity of AC mainly limited the clinical application of AC-containing herbs and preparations. In order to prevent clinical AC poisoning, doctors usually use processed herbs instead of the raw herbs. The AC content of processed herbs is close related to clinical efficacy and safety because AC is the main pharmacodynamic and toxic component in these herbs. Singhuber et al. and Wada et al. reported that the LD_50_ values of AC in mice were 1.8 mg/kg p. o., 0.31 mg/kg i. p., 0.12 mg/kg i. v., and 0.27 mg/kg i. p. (Wada et al., 2004; Singhuber et al., 2009). The human lowest lethal dose was 28 mg/kg bodyweight (Song et al., 2001). AC and related alkaloids have been proved to be highly cardiotoxic and neurotoxic. The toxic mechanism was related to the actions of AC on the voltage-sensitive sodium channels of the cell membranes of excitable tissues, including the myocardium, nerves, and muscles (Chan, 2009). The presence of extracellular sodium, induced by AC, caused the reduced inactivation of the alpha subunits of the human heart (hH1) channels (Wright, 2002). Wang et al. reported that AC possessed binding stability for the receptor calcium-calmodulin–dependent protein kinase gamma (CAMK2G) (Wang et al., 2018). Sun’s results indicated that AC significantly aggravated Ca^2+^ overload and caused arrhythmia and finally promotes apoptotic development *via* phosphorylation of P38 mitogen-activated protein kinase (Sun et al., 2014). Also, AC could induce cardiomyocyte damage by mitigating BNIP3-dependent mitophagy and the TNFα-NLRP3 signaling axis (Peng et al., 2019). Ye et al. suggested that AC might induce cardiotoxicity in zebrafish by activating Na^+^ channel and inhibiting K^+^ channel (Ye et al., 2019). Liu et al. reported that AC induced cardiotoxicity and apoptosis in embryonic zebrafish by influencing the expression of cardiovascular relative genes, such as *Tbx5*, *Gata4*, and *Nkx2.5* (Liu et al., 2019). Moreover, apoptosis was also observed in Liu’s research (Liu et al., 2019). Li et al. confirmed that AC induced cardiac dysfunction and apoptosis through the regulation of the calcium signaling pathway in zebrafish and H9c2 cells (Li et al., 2020a). In traditional Chinese medicine, raw *Aconitum* is strictly forbidden for pregnant women. Furthermore, pregnant women should avoid processed *Aconitum* administration except for the physician’s declaration. However, the understanding of the developmental toxicity of AC is limited. Li et al. showed that aconitine induces a concentration-dependent mortality of zebrafish embryos (Li et al., 2020a). Mortality is also a sign of toxicity. Furthermore, an increase in coiling frequency, also a sign of embryotoxicity, has been reported recently. Wang et al. found that AC-induced cardiotoxicity in zebrafish predominantly included arrhythmias, extended sinus venous and bulbus arteriosus (SV-BA) distance, and larger pericardial edema aera (Wang et al., 2020). So far only one report had been published and showed that AC had direct embryotoxic effects during the rat organogenetic period (Xiao et al., 2007). A more detailed assessment of the developmental toxicity and underlying mechanisms of AC is required.

Zebrafish has arisen as a popular alternative animal model and provides 3Rs value to drug discovery toxicology (Cassar et al., 2020). Conserved vertebrate biology, ease of husbandry, high fecundity, small size, rapid development, and transparent young are some of the main attractions of zebrafish as an alternative to mammals for toxicology studies (Xia et al., 2018; Song et al., 2020).

In the present study, a developmental toxicity assay of AC was performed using AB strain and transgenic zebrafish line embryos for the first time. The toxic effects of AC on embryonic mortality, hatching rate, malformations, and their target organ toxicity assay on developing heart, liver, and nervous system were tested. The oxidative stress, cell apoptosis, Nrf2/HO-1, and JNK/Erk pathways signaling-related genes expression levels were also assessed. Taken together, this study provides valuable insights of AC-induced developmental toxicity, as well as the underlying molecular mechanisms.

## Materials and Methods

### Chemicals

AC (CAS number 302-27-2, purity ≥98.0%) was purchased from Chengdu DeSiTe Biological Technology Co. Ltd (Chengdu, Sichuan, China). A stock solution (20 mM) of AC was prepared in dimethyl sulfoxide (DMSO), aliquoted, and stored at 4°C until use. The working solutions were obtained by diluting the stock solution with embryo water (5 mM NaCl, 0.17 mM KCl, 0.4 mM CaCl_2_, and 0.16 mM MgSO_4_) before the experiments. All other chemicals and reagents used in this study were of analytical grade.

### Zebrafish Maintenance and Egg Collection

Zebrafish lines used in this study were obtained from the Key Laboratory of Drug Screening Technology, Biology Institute (Jinan, Shandong, China). The AB strain zebrafish (*Danio rerio*), *Tg(myl7:EGFP)*, *Tg(L-FABP:EGFP)*, and *Tg(Vmat:GFP)* transgenic zebrafish lines were maintained under a constant 14 h light/10 h dark cycle photoperiod at 28 ± 0.5°C in an automatic zebrafish housing system (ESEN, Beijing, China). Adult fish were fed live brine shrimp twice a day. The adult zebrafish were placed into mating tanks with a transparent divider in the evening. The next morning, the divider was pulled at lights on, and the fish were allowed to natural spawning. After 2 h, eggs were collected and kept in embryo water containing 2 mg/l methylene blue. At 4 h post fertilization (hpf), the embryos were examined under a stereomicroscope (SZX16, Olympus Tokyo, Japan), and those that had developed normally to the blastula stage were chosen for subsequent experiments. All experiments were carried out in compliance with the standard ethical guidelines and under the control of the Biology Institute, Qilu University of Technology of Animal Ethics Committee.

### Lethal and Teratogenicity Assay

The normal embryos at 4 hpf were randomly distributed into 24-well plates (10 larvae per well) and exposed to various concentrations of AC (4, 6, 8, 10, 12, 14, 16, or 18 μM) diluted in 2 ml of embryo water. Zebrafish embryos treated with 0.1% DMSO were considered vehicle controls. The exposure solutions were changed every 24 h. Meanwhile, dead embryos were removed and recorded. The exposure began at approximately 4 hpf and ended at approximately 96 hpf. The values corresponding to the 10% lethal concentration (LC_10_) and 1% lethal concentration (LC_1_) were calculated based on 96 h dose-response curves. A series of concentrations at low lethal and none lethal range were selected to teratogenic assays, to avoid the common injury induced by death. In this study, 0.73 (1/10 LC_1_), 2.42 (1/3 LC_1_), 7.27 (LC_1_), and 8.23 (LC_10_) μM were used in the following assays. Bright-field images of the embryos were photographed using a fluorescence stereomicroscope (AXIO Zoom. V16, ZEISS, Oberkochen, Germany) at 24, 48, 72, and 96 hpf. The hatching rate of embryos at 72 hpf was calculated. The embryonic developmental parameters for body length (head-to-tail axis), mortality, and malformation rate at 96 hpf were also recorded.

### Assessment of the Effect of AC on the Developing Heart

The *Tg(myl7:EGFP)* zebrafish embryos, which express specific fluorescence in the myocardial cells, were used to evaluate the effect of AC on the developing heart. After immobilized in 4% methyl cellulose, heart phenotypic lateral view images of zebrafish embryo at 72 and 96 hpf were obtained using bright-field and fluorescence stereomicroscope (ZEISS). The pericardial area of each embryo was measured using the Image-Pro Plus software (Media Cybernetics, Bethesda, MD, United States). A 20 s bottom view fluorescence video of zebrafish heartbeats were taken at 72 and 96 hpf under the fluorescence stereomicroscope. The heartbeats of 10 zebrafish from each group were counted manually in a 20 s period, which was multiplied by three to calculate heart rate. Images of ventricular end-diastolic and ventricular end-systolic phases of ventricle were selected from the video, to measure ejection fraction and stroke output. Pericardial area and SV-BA distance were measured from bright-field images.

### Assessment of the Effect of AC on the Developing Liver

The transgenic zebrafish *Tg(L-FABP:EGFP)* embryos that expressed liver-specific fluorescence in the liver were used in this study. The liver toxicity was assessed by the size of the liver area and fluorescence intensity. At 96 hpf, lateral view images of the liver were measured in both control and AC-treated groups using the fluorescence stereomicroscope. The area and fluorescence intensity of the liver tissue were determined using the Image-Pro Plus software.

### Assessment of the Effect of AC on the Developing Nervous System

The transgenic zebrafish *Tg(Vmat:GFP)* embryos that expressed green fluorescent protein in the dopaminergic neurons were photographed from the dorsal view using the fluorescence stereomicroscope. The length of the dopamine ganglion was measured using the Image-Pro Plus software. Furthermore, 10 larvae of each group were randomly transferred to a 48-well plate (1 larva per well) at 120 hpf and 30 min of the motion trail of each larva was recorded using zebralab (Viewpoint, Lyon, France), followed by the measurement of the total swimming distance and swimming velocity of each fish over the 30 min. The swimming behavior of zebrafish at 120 hpf is more repetitive than that at 96 hpf, so we did not detect behavior until 120 hpf.

### Measurement of ROS Generation

The ROS content was measured using a fluorescent probe DCFH-DA as a probe for reactive oxygen detection in AB strain zebrafish. After treatment with AC, zebrafish embryos at 96 hpf were rinsed twice with PBS and incubated with 30 μM DCFH-DA for 40 min in the dark at 28 ± 0.5°C. Then, the embryos were rinsed three times with PBS. The lateral view images of each embryo were taken using the fluorescence stereomicroscope. The fluorescence intensity of individual embryos was quantified using the Image-Pro Plus software.

### Measurement of T-SOD Activity and Lipid Peroxidation

At 96 hpf, 50 zebrafish embryos were pooled together and rinsed with embryo water. Thereafter, the embryos were collected to a centrifuge tube and homogenized on ice cold with 500 μl saline. Briefly, the embryo homogenate was centrifuged at 2,500 rpm for 10 min at 4°C. After supernatants collection, total superoxide dismutase (SOD) enzyme activities and malondialdehyde (MDA) levels were estimated using commercial kits (Beyotime Biotechnology) following the manufacturer’s protocols. The T-SOD kit is a color reaction based on WST-8, and the absorbance was measured at 450 nm. The MDA kit is designed using the principle of MDA and thiobarbituric acid reaction to produce red products. The absorbance was measured at 535 nm.

### Acridine Orange Staining

This method was described in our previous article (Song et al., 2020). Briefly, after AC treatment, zebrafish embryos at 96 hpf were rinsed twice with PBS and incubated with 10 μg/ml AO in PBS for 20 min in the dark at room temperature. Twenty minutes later, the embryos were thoroughly washed with PBS three times. Microscopy evaluation was performed on 0.16% tricaine (Sigma Aldrich, St. Louis, MO, United States) anesthetized larvae by using the fluorescence stereomicroscope.

### Terminal Deoxynucleotide Transferase-Mediated UTPnick End Labeling Assay

This method was described in our previous article (Song et al., 2020). Briefly, the apoptosis in AC-treated embryos was detected using the One Step TUNEL Apoptosis Assay Kit (Beyotime Biotechnology). Briefly, the embryos were fixed in 4% paraformaldehyde (PFA) at 4°C overnight. After washing with PBS, endogenous peroxidases were blocked by incubation in 3% hydrogen peroxide in methanol for 15 min at room temperature. The larvae were rinsed twice with PBS and followed by incubation with TUNEL reaction mixture at 37°C for 60 min. After washing with PBS twice, the larvae were photographed by using the fluorescence stereomicroscope.

### Real-Time Quantitative PCR

This method was described in our previous article (Song et al., 2020). Briefly, the total RNA was extracted from 96 hpf embryos (*n* = 40) using Fastpure cell/Tissue Total RNA Isolation Kit (Vazyme Biotech Company Ltd., Nanjing, Jiangsu, China) following the manufacturer’s protocol. Briefly, cDNA was generated using a HiScript II QRT SuperMix for qPCR (+gDNA wiper) (Vazyme Biotech). qPCR was performed using an AceQ qPCR SYBR Green Master Mix (Vazyme Biotech) and the Light Cycler® 96 System (Light Cycler® Instrument; Roche; Switzerland). Runs were carried out in triplicate using the housekeeping gene *rpl13a* to normalize the mRNA levels of target genes. Primer sequences are available on request.

### Statistical Analyses

All data are presented as mean ± standard error (SD). All data were checked for normality first. The significance of data that fit a normal distribution was determined by using one-way analysis of variance (ANOVA) followed by Dunnett’s post-hoc test using GraphPad Prism 6 (La Jolla, CA, United States). The significance of data that did not fit a normal distribution was analyzed using Kruskal–Wallis analysis by GraphPad Prism 6. Significant differences compared with the control were identified when the *p* value was less than 0.05 or 0.01. All the experiments were performed in triplicate.

## Results

### Lethality and Teratogenicity of AC in Zebrafish Embryos

**FIGURE 1:**
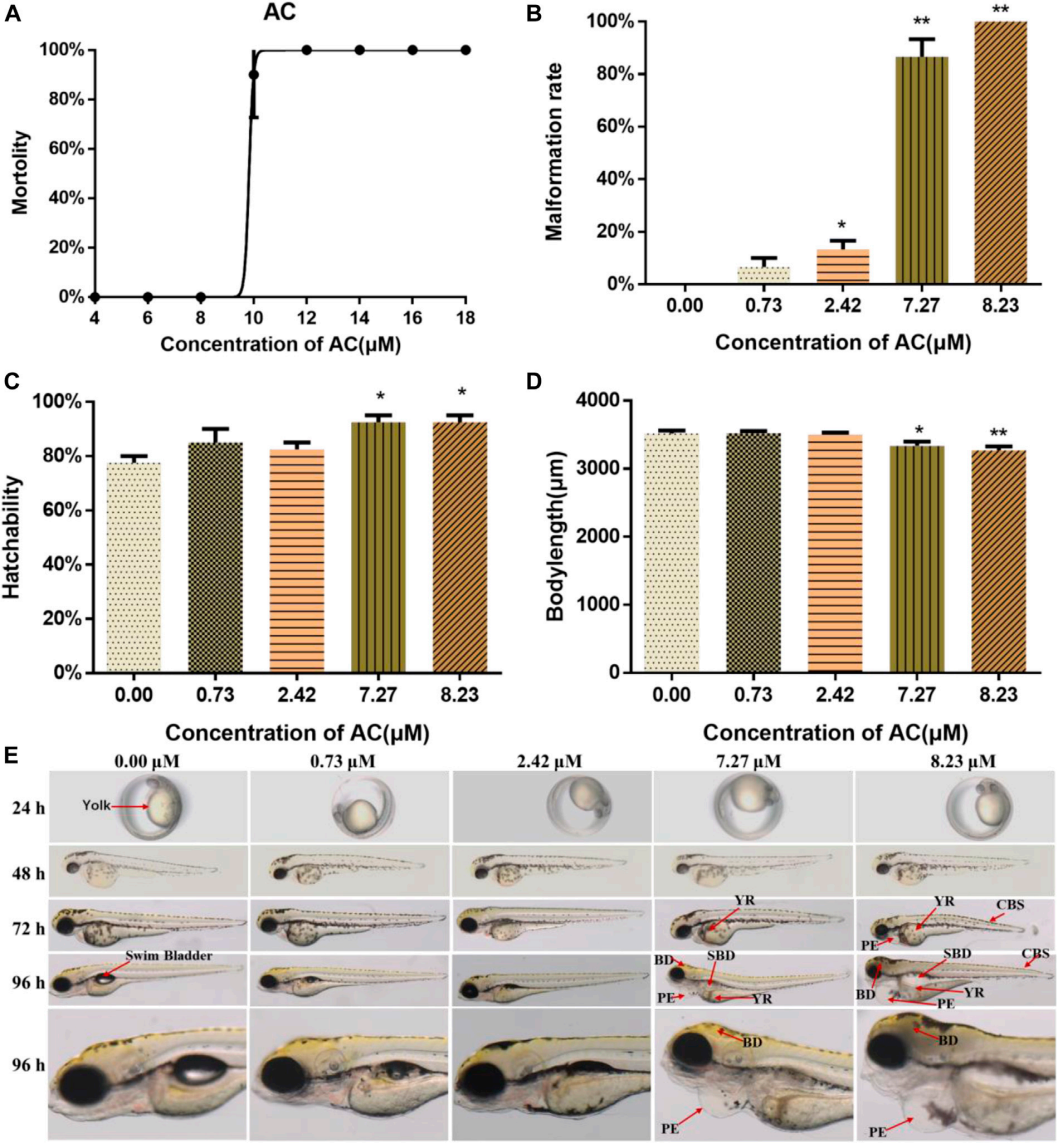
Lethal and teratogenic effects of AC in zebrafish larvae. **(A)** Mortality-concentration curve. **(B)** Malformation rates at 96 hpf. **(C)** Hatchability of embryos at 72 hpf. **(D)** Body length at 96 hpf. **(E)** Teratogenic effects of AC on embryo phenotype from 24 to 96 hpf. BD, brain deficiency; CBS, curved body shape; PE, pericardial edema; SBD, swim bladder deficiency; YR, yolk retention. The values are expressed as mean ± SD (*n* = 3, which means three pools of 10 larvae). *p < 0.05 and **p < 0.01 vs. control.

The lethal curve was shown in Figure 1A. According to our assessment, the LC_10_ and LC_1_ values at 96 hpf were calculated as 8.23 and 7.27 μM, respectively. To investigate the possible developmental teratogenic effects of AC on zebrafish embryos, mortality rate, malformation rate, hatching rate, body length, and morphological abnormalities were recorded at 24, 48, 72, and 96 hpf. AC exposure caused teratogenicity and lethal toxicity in zebrafish embryos especially in 7.27 and 8.23 μM treated groups. In the developmental toxicity assay, we found AC induced significant morphological phenotypes, most prominently yolk retention, swim-bladder deficiency, pericardial edema, and curved body shape, in a dose-dependent manner (Figures 1B,E). As compared with the control group, both 7.27 and 8.23 μM of AC-treated groups showed significantly higher malformation rates. The malformation rate reached 100% at 8.23 μM AC (Figure 1B). The hatchability at 72 hpf was significantly increased especially on 7.27 and 8.23 μM AC treatment (Figure 1C). The body length of embryos (*n* = 30) was significantly reduced in a concentration-dependent manner at 96 hpf, especially in the 7.27 and 8.23 μM AC-treated groups as compared with the control (Figure 1D).

As shown in Figure 1E, the malformation was first noticed at 72 hpf. Compared with the embryos in the control group, both 7.27 and 8.23 μM of AC-exposure embryos showed severe pericardial edema, swim bladder deficiency, yolk retention, brain deficiency, and curved body shape (Figure 1E).

### AC Induces Cardiac Developmental Toxicity

**FIGURE 2:**
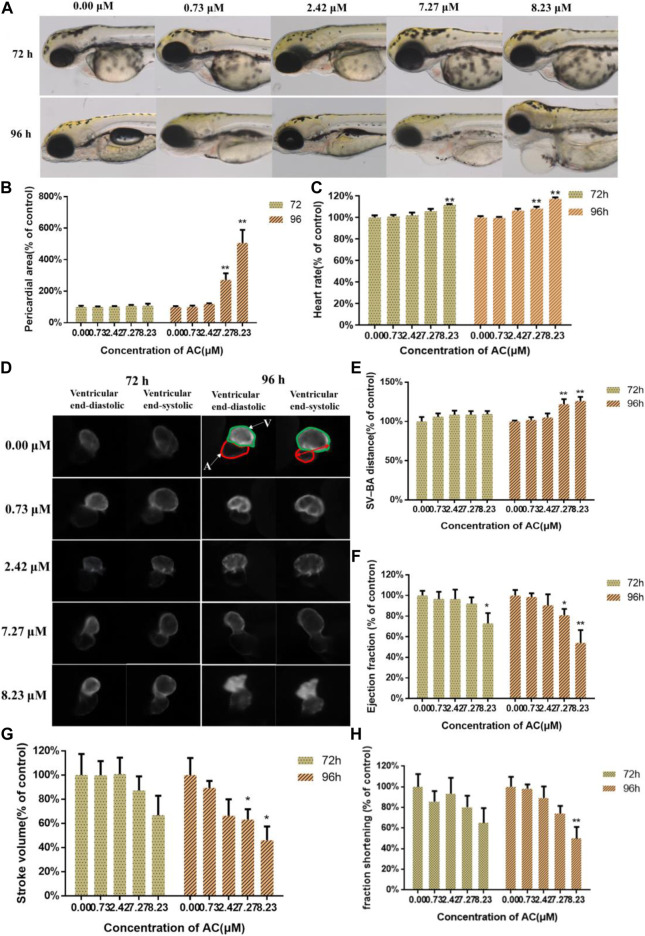
Effects of AC on developing heart. **(A)** Bright-field images of the *Tg(myl7:EGFP)* zebrafish embryos at 72 and 96 hpf. A, atrium; V, ventricle. The red double-headed arrow represents SV-BA distance. **(B)** Pericardial area of embryos at 72 and 96 hpf. **(C)** The heart rate of embryos at 72 and 96 hpf. **(D)** The images of ventricular end-diastolic and end-systolic at 72 and 96 hpf. **(E)** SV-BA distance of embryos at 72 and 96 hpf. **(F)** Ejection fraction of embryos at 72 and 96 hpf. **(G)** The stroke volume of embryos at 72 and 96 hpf. **(H)** Fraction shortening of embryos at 72 and 96 hpf. The values are expressed as mean ± SD (*n* = 10). *p < 0.05 and **p < 0.01 vs. control.

The phenotype of zebrafish embryos at 72 and 96 hpf were shown in Figure 2A. When assessing the morphology of zebrafish embryo after AC treatment, the most obvious malformation was observed as pericardial edema in 7.27 and 8.23 μM treated groups at 96 hpf. Compared to the control group, the pericardial areas at 96 hpf (Figure 2B) and heartbeats at 96 hpf were significantly increased in a dose-dependent manner (Figure 2C), especially with 7.27 and 8.23 μM AC-treated groups. The heart rates of 76 hpf embryos (Figure 2C) showed a significant increase in 7.27 and 8.23 μM AC-treated groups as compared with the control. Figure 2D shows bottom view images of the ventricular at the end-diastolic and end-systolic phases at 72 and 96 hpf. The SV-BA distance of embryos at 96 hpf in the 7.27 and 8.23 μM groups was significantly increased (Figure 2E). The ejection fraction of embryo ventricular at 72 hpf in the 8.23 μM AC-treated group was significantly decreased (Figure 2F). At 96 hpf, the ejection fraction was decreased both in 7.27 and 8.23 μM of AC-treated embryos (Figure 2F). The stroke volume and fraction shortening at 96 hpf were significantly decreased in a dose-dependent manner, respectively. The stroke volume in 7.27 and 8.23 μM of AC-treated embryos at 96 hpf was significantly decreased (Figure 2G). The fraction shortening in 7.27 and 8.23 μM of AC-treated groups at 96 hpf was significantly decreased (Figure 2H).

### Effects of AC on Developing Liver

**FIGURE 3:**
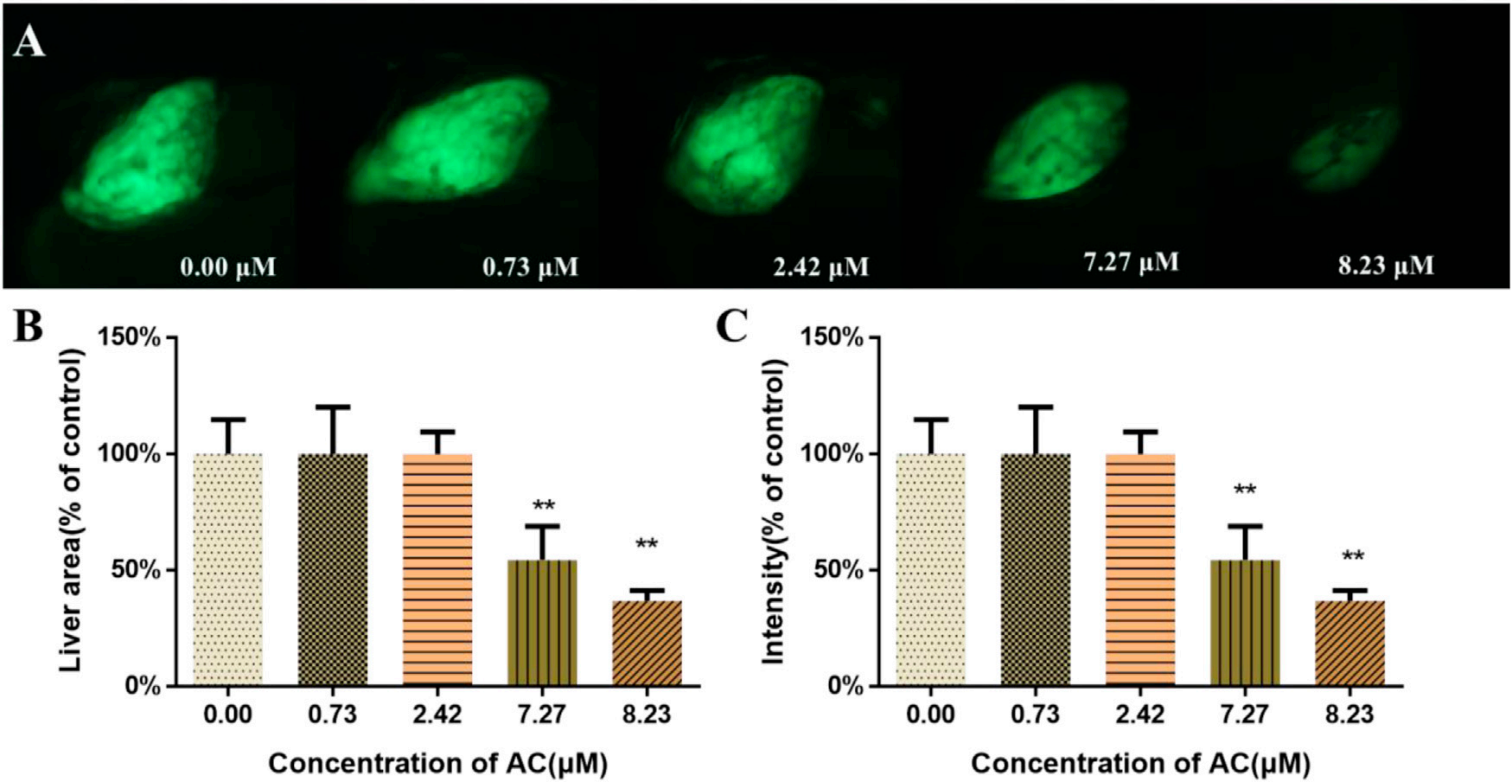
Effects of AC on developing liver. **(A)** The lateral view images of developing livers of *Tg(L-FABP:EGFP)* zebrafish embryos, which express a *green fluorescent* protein (*GFP*) in the liver at 96 hpf. **(B)** Liver area of embryos at 96 hpf. **(C)** The liver intensity of embryos at 96 hpf. The values are expressed as mean ± SD (*n* = 10). *p < 0.05 and **p < 0.01 vs. control.

To assess the toxicity of AC on the development of zebrafish liver, *Tg(l-fabp-EGFP)* transgenic zebrafish embryos were used. As shown in Figure 3A, AC exposure exhibited toxic effects on liver development. AC treatment decreased the liver area and intensity both in a dose-dependent manner. The liver area and fluorescence intensity of AC-treated embryos were significantly diminished, especially in higher concentrations of 7.27 and 8.23 μM AC-treated groups than that in the control group (Figures 3B,C).

### Effects of AC on Developing Nervous System

**FIGURE 4:**
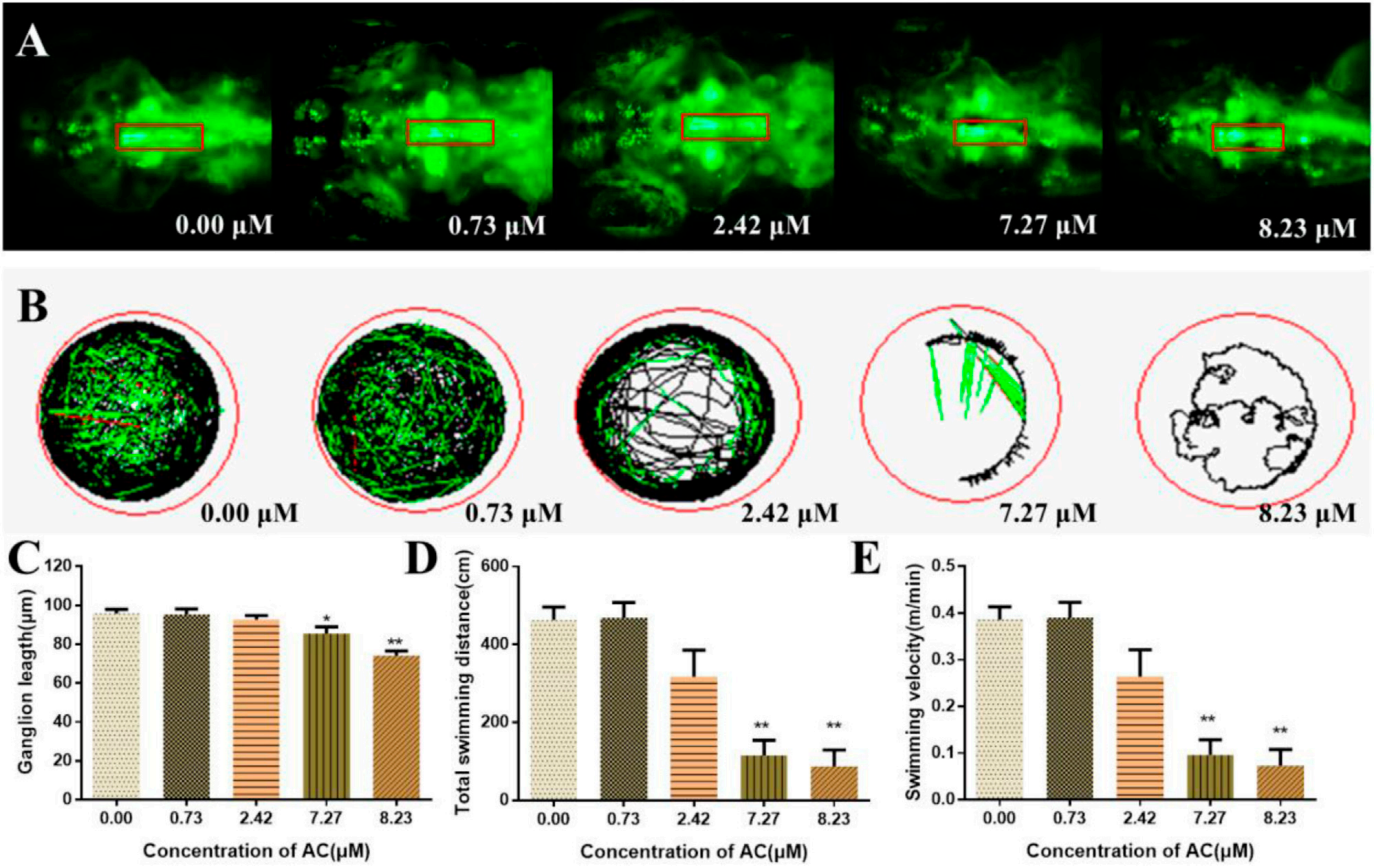
Effects of AC on developing nervous systems. **(A)** Dorsal view fluorescence images of 96 hpf *Tg(Vmat:GFP)* embryos show dopaminergic neurons of brain. The red box represents the raphe nuclei cluster of dopaminergic/vesicular monoamine transporter 2–positive neurons. **(B)** Tracking images of larval swimming activity reduced gradually with the doses increasing at 7 dpf. **(C)** Length of dopamine ganglion at 96 hpf. **(D)** Total swimming distance of embryos at seven dpf. **(E)** Swimming velocity of embryos at 7 dpf. Values are expressed as mean ± SD (*n* = 10). *p < 0.05 and **p < 0.01 vs. control.

To examine whether AC exposure could affect the neural development in the zebrafish brain, we used a transgenic line expressing a green fluorescent protein on the dopaminergic/vesicular monoamine transporter 2-positive neurons (Figure 4A). As shown in Figures 4A,C, AC exposure induced the loss of dopaminergic neurons of raphe nuclei cluster on zebrafish embryo. The length of the dopaminergic neurons of the raphe nuclei cluster was significantly reduced as the exposure dosages increased, especially in 7.27 and 8.23 μM of AC-treated groups. The tracking images of larval locomotor activity of embryos were shown in Figure 4B. The total swimming distance and swimming velocity were significantly decreased (Figures 4D,F), especially in the 7.27 and 8.23 μM of AC-treated embryos as compared with the control group.

### ROS, T-SOD, and MDA Measurement

**FIGURE 5:**
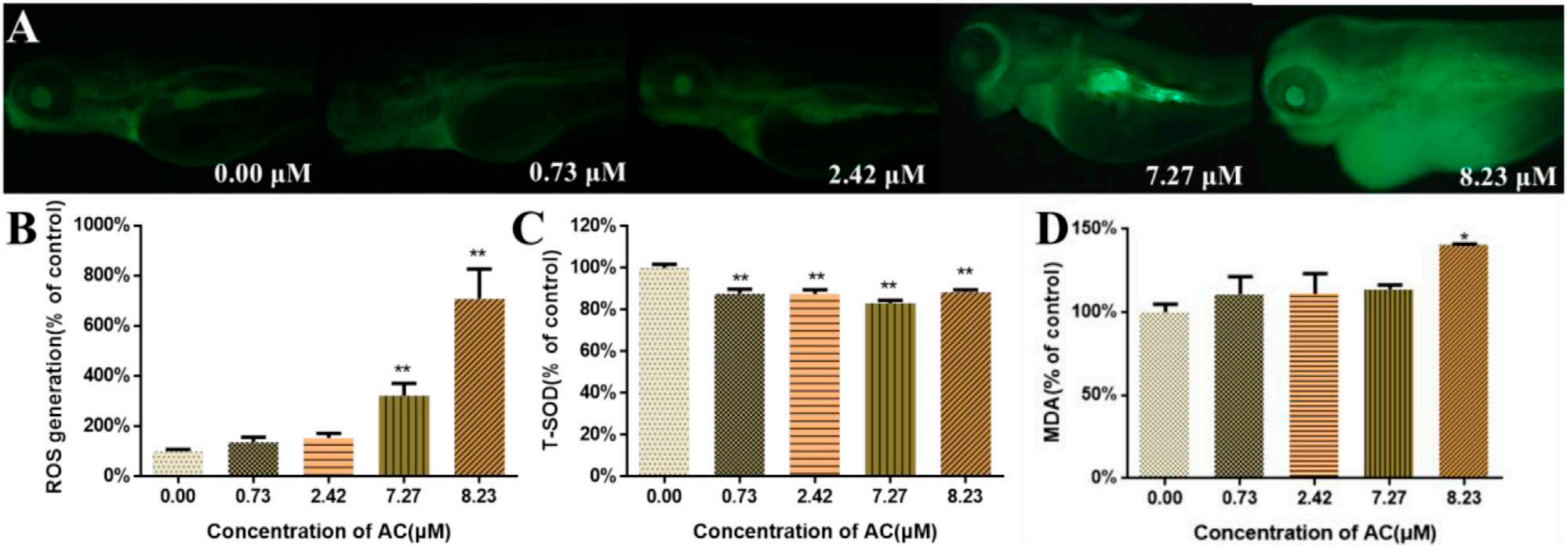
ROS, T-SOD, and MDA measurement. **(A)** Images of zebrafish larvae subject to ROS assay in fluorescence field at 96 hpf. **(B)** ROS generation at 96 hpf (*n* = 10). **(C)** The activities of T-SOD at 96 hpf (*n* = 3, which means 3 pools of 50 larvae). **(D)** The content of MDA at 96 hpf (*n* = 3, which means 3 pools of 50 larvae). Values are expressed as mean ± SD. *p < 0.05 and **p < 0.01 vs. control.

As shown in Figures 5A,B, the ROS generation in AC-treated zebrafish was increased in a dose-dependent manner. Notably, in 7.27 and 8.23 μM AC-treated groups, the ROS content was ∼3 and ∼6 times higher than that in the control group. After AC treatment, the effects of AC on SOD and MDA in larvae were detected (*n* = 3 pools, 50 embryos per pool). The results showed that the activities of SOD significantly decreased in AC-treated embryos (Figure 5C), whereas the MDA levels were significantly increased in 7.27 and 8.23 µM AC-treated groups (Figure 5D).

### Apoptosis Detection

**FIGURE 6:**
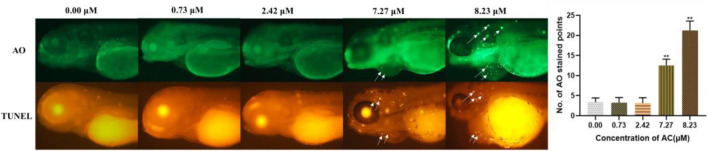
AC-induced apoptosis in 96 hpf zebrafish embryo. AO and TUNEL staining of the apoptotic cells (white arrows indicate apoptotic cells in the brain and heart).

To investigate the developmental toxicity of AC in programmed cell death, AO and TUNEL staining of live embryos were performed. As shown in Figure 6, the incidence of the apoptotic cells was significantly increased in the heart and brain regions of 7.27 and 8.23 μM AC-treated embryos, whereas there were no apoptotic cells observed in the lower concentration of the AC-treated group. The results of acridine orange staining were consistent with those of TUNEL assays. These results correlated with the incidence of malformations, which indicated that brain regions, pericardia, and the heart region were more sensitive to AC.

### Effects of AC on Gene Expression

**FIGURE 7:**
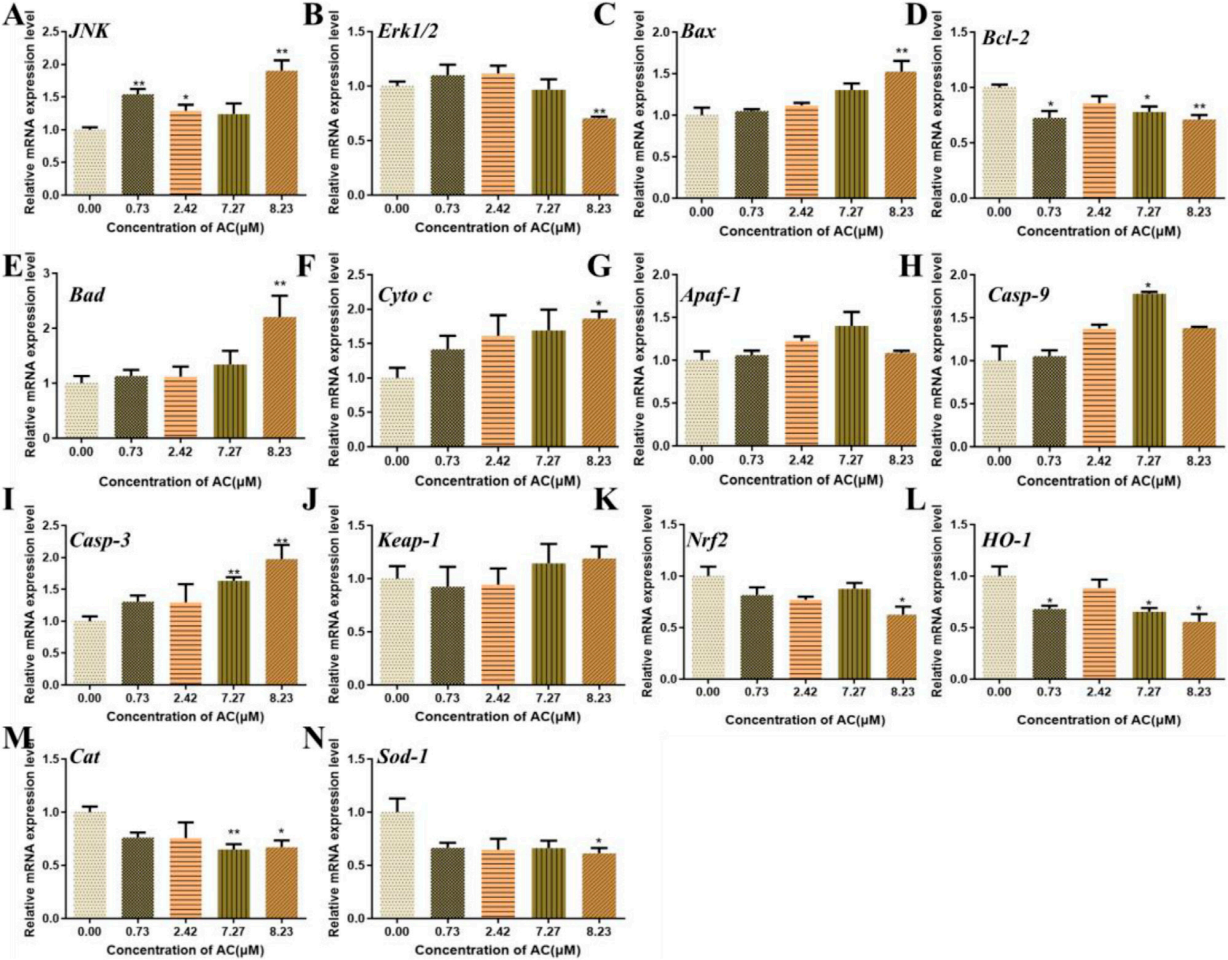
Effects of AC on the expression of oxidative stress, *JNK*, and mitochondrial apoptosis-related genes in zebrafish larvae at 96 hpf.

To investigate the possible molecular mechanisms of AC-induced developmental toxicity, the relative mRNA expression of various genes related to oxidative stress and apoptosis were detected using real-time quantitative PCR. As shown in Figure 7, the expression of *JNK* was upregulated, compared with the control. The expression of *Erk1/2* was significantly downregulated in the 8.23 μM AC-treated group. The expressions of apoptosis-related genes (*Bax*, *Bad*, *Cytochrome C*, *Caspase-9*, and *Caspase-3*) were upregulated. The mRNA expression level of *Bcl-2* was significantly downregulated. Besides, the expressions of genes related to oxidative stress (*Nrf2*, *HO-1*, *Cat*, and *Sod-1*) were downregulated. However, the expression of *Apaf-1* and *Keap-1* were not significantly changed in the AC-treated groups compared to the control.

The mRNA levels of *JNK* (A), *Erk1/2* (B), *Bax* (C), *Bcl-2* (D), *Bad* (E), *Cyto C* (F), *Apaf-1* (G), *Casp-9* (H), *Casp-3* (I), *Keap-1* (J), *Nrf2* (K), *HO-1* (L), *Cat* (M), and *Sod-1* (N). The values are expressed as mean ± SD (*n* = 3, which means three pools of 40 larvae). *p < 0.05 and **p < 0.01 vs. control.

## Discussion

AC is one of the effective ingredients of diester-diterpene alkaloids extracted from *Aconitum* plant roots and plays an important role in the bioactivities of analgesic, cardiotonic, antitumor, anti-asthma, and anti-inflammatory agents (Borcsa et al., 2010; Tang et al., 2012; Nyirimigabo et al., 2015; Ji et al., 2016). The improper use of AC in traditional herbal medicine possesses a high risk of toxicities associated with the cardiovascular and nervous system. Several studies have demonstrated that AC at various concentrations induced cardiac dysfunction, apoptosis, and toxicity-mediated Ca^2+^ signaling mechanisms in zebrafish embryos (Liu et al., 2019; Li et al., 2020a). Although the cardiotoxicity and neurotoxicity of AC have been widely reported by various scientific studies, still there is lack of available information on the detailed underlying mechanism of AC effect on embryonic development. Here we aimed to study the toxic effects of AC on development of the zebrafish embryos/larvae. Our results showed that AC significantly affected the embryonic development of the zebrafish larvae, especially in the heart, brain, and liver. Further studies showed that the generation of ROS, oxidative stress, and subsequent activation of mitochondrial apoptosis mediated by Nrf2/HO-1 and JNK/Erk signaling pathways might be the underlying mechanism of the developmental toxicity induced by AC.

In the present study, our findings demonstrated that AC exposure in zebrafish led to embryonic developmental toxicity and increased malformation rates including suppressed body length, curved body shape, pericardial edema, and brain defects. Similar to our results, Xiao and coworkers reported the toxic effects of AC on the embryonic development of rat embryos (Xiao et al., 2007). The reduced crown-rump length and head length, irregular somites, lower morphologic score, cardiac defect (undivided cardiac tube and inflated pericardial cavity), and brain malformation were observed in embryos at >2.5 μg/ml (3.87 μM) AC treatment (Xiao et al., 2007). During the AC exposure period, we observed unique developmental malformations appear at different endpoints. Yolk retention appeared at 72 hpf. As the AC exposure dosages continued to 96 hpf, swim bladder defects were further identified. The most pronounced phenotypes were observed as pericardial edema in AC treated embryos, which occurs in a dose-dependent manner. These data briefly showed that AC induced developmental defects and affect the embryonic development of zebrafish. The present article and Xiao’s reports (Xiao et al., 2007) both proved that AC had direct developmental toxicity to embryos. The placenta plays a significant role in drug distribution in the fetus. However, zebrafish embryos develop *in vitro* do not have blood-placental barrier. Liposoluble AC may be transported through the placenta to interfere with the growth and development of embryos. Nevertheless, the effects of AC on placental function and the placental transfer of AC still need to be detected.

Cardiac dysfunction and cardiac malformations are the major toxic effects of AC, which has been caused by their actions on the voltage-sensitive sodium channels of the cell membranes of excitable tissues, including the myocardium, nerves, and muscles (Wright, 2002; Sun et al., 2014; Peng et al., 2019; Li et al., 2020a). To assess the cardiotoxicity of AC on zebrafish embryo/larvae, we used *Tg(myl7:EGFP)* zebrafish embryos which express an enhanced green fluorescent protein on myocardial progenitors and cardiomyocytes (Zhen et al., 2012). Our study showed that AC induced slight heart developmental toxicities in *Tg(myl7:EGFP)* embryos by increasing heart rate and decreasing ejection fraction at 72 hpf. Further, the data at 96 hpf showed that AC induced severe heart developmental toxicity in a dose-dependent manner. Li et al. evaluated the AC-induced cardiotoxicity on zebrafish from 48 to 96 hpf and measured the heart rated every 12 h (Li et al., 2020a). They found that AC exposure decreased zebrafish heart rate, which is contrary to our results. There are two possible reasons for this. The first one is fish age difference. In our study, the zebrafish is exposure to AC from 4 to 96 hpf. Li et al. used zebrafish at 48 hpf. More important is the second reason. According to cardiotoxicity evaluation methods on zebrafish, Li et al. might remove the embryos membrane manually, which made the frail young embryos directly exposure to AC solution, leading to high toxic effects on heartbeats. The pericardial area, heart rate, and SV-BA distance were significantly increased. The ejection fraction, stroke volume, and fraction shortening were decreased. In addition to this, we found that AC promoted the apoptotic response of hearts *in vivo*. We performed AO staining and TUNEL assay to identify apoptosis in AC-treated embryos. The consistent finding of both AO and TUNEL staining revealed that the huge number of apoptotic cells was predominantly localized in the heart and brain region. A similar observation has also been reported on recent studies that AC at various concentrations induced apoptosis and cardiac dysfunction in zebrafish embryos (Liu et al., 2019; Li et al., 2020a). In keeping with our apoptotic results, we also noted that pericardial edema was one of the most obvious phenotypes in AC-treated groups. Thus, our results could also confirm that the zebrafish heart might be more sensitive to AC-induced developmental toxicity.

To better understand the obvious signs of malformation reported in Figure 1, the target organ toxicity assays were performed by using transgenic zebrafish lines express with GFP in the liver, heart, and CNS. So it can be easy and direct to record the toxic effects of AC on different organs or certain types of cells at the different developmental stages of the embryos under the microscope. Zebrafish are similar to humans in hepatic cellular composition, function, signaling, and response to injury as well as the cellular processes that mediate liver diseases (Goessling and Sadler, 2015). In this study, the shrinkage of the liver area and their fluorescence intensity manifested that AC exhibited toxic effects on liver development. The development processes and mechanisms of the central nervous system, including the blood-brain barrier, of zebrafish and other vertebrates are well conserved (d’Amora and Giordani, 2018). Neural morphogenesis and neurobehavioral profiling are regarded as important endpoints to evaluate the toxic effects of chemicals on the developing nervous system (Miller et al., 2018). Our data showed that AC treatment decreased the length of the dopaminergic neurons, total swimming distance, and velocity, which indicated that AC processed toxic effects on zebrafish developing nervous system. Taken together, these results indicated that AC significantly affects the development and function of the liver and nervous system of the zebrafish embryo.

However, little is known regarding the molecular mechanisms of AC-induced developmental toxicity. Oxidative stress is generally defined as an imbalance that favors the production of ROS over antioxidant defenses (Orrenius et al., 2007). Our data showed that AC treatment led to a significant increase in ROS-mediated toxicity in embryos. Moreover, the T-SOD activity was decreased and MDA content was increased. These results demonstrated that oxidative stress had occurred in AC-treated zebrafish. ROS can cause lipid modification, DNA damage, and protein damage which can lead to the activation of different modes of cell death, including apoptosis (Orrenius et al., 2003). Besides ROS generation, AC also triggered cell apoptosis mainly at the heart and brain at higher concentrations (Figure 6). Hence, it can be speculated that the ROS-medicated mitochondrial apoptosis was the molecular mechanism of AC-induced developmental toxicity.

Oxidative stress-medicated apoptosis is implicated in a variety of drug-induced toxicities (Deavall et al., 2012). The multifunctional regulator nuclear factor erythroid 2-related factor (Nrf2) is considered as a cytoprotective factor regulating the expression of genes coding for anti-oxidant (Loboda et al., 2016). The major characteristics of Nrf2 are to some extent mimicked by Nrf2-dependent genes and their proteins including heme oxygenase-1 (HO-1). HO-1, by regulating intracellular levels of pro-oxidant heme, had become an important candidate protein to be upregulated to combat oxidative stress (Waza et al., 2018). Additionally, it is well established that HO-1-deficiency results in embryonic death, and some studies evaluated the role of HO-1 in embryonic survival (Loboda et al., 2016). Excessive generation of ROS in cells leads to activation of MAPK pathways, including ERKs, JNKs, or p38 MAPKs (Thongsom et al., 2017). ROS activated JNK phosphorylation in berberine- and free fatty acid-induced SW620 cells triggered bovine hepatocytes apoptosis and downregulated ERK phosphorylation in response to 4-OHE2–induced cell death (Li et al., 2020b). The changes in JNK and ERK that are induced by many stimuli are involved in the transcription or phosphorylation of Nrf2 (Cheng et al., 2008; Sun et al., 2009). Moreover, oxidative stress-associated JNK/ERK-mediated apoptosis requires inhibition of the transcription factor Nrf2 in various cell types (Li et al., 2020b).

To further study the underlying mechanism of developmental toxicity induced by AC, the expression levels of several genes related to oxidative stress and apoptosis were measured. The nuclear factor erythroid 2-related factor 2 (Nrf2) is an emerging regulator of cellular resistance to oxidants. Nrf2 regulates many of the antioxidant enzymes/proteins to hold redox signaling in the local environment (Ma, 2013). Heme oxygenase-1 (HO-1) and its products exert beneficial effects against oxidative injury and regulation of apoptosis (Loboda et al., 2016). In the present study, the expression levels of *Nrf2*, *HO-1*, *Cat*, and *Sod-1* were significantly downregulated, which indicated that the antioxidant capacity of cells was extremely reduced. However, no significant change of *keap-1* expression was observed in our study. Under normoxic condition, KEAP1 binds to Nrf2 in the cytoplasm. Under oxidative stress stimulation, free Nrf2 enters nucleus and activates the transcription of downstream genes to protect cells from oxidative stress. Our results showed that *keap-1* expression was not significantly changed while the *nrf2* expression was downregulated, which might have resulted in no free Nrf2 in the cytoplasm leading to lack of antioxidant capacity. Extracellular signal-regulated kinases (Erk1/2) and c-Jun NH2-terminal kinase (JNK) is a common requirement for caspase activation (Sinha et al., 2013). ROS is a major regulator of JNK activation (Kamata et al., 2005). In this study, the expression level of *JNK* was upregulated and the expression level of *Erk1/2* was downregulated. The changes in *JNK* and *Erk1/2* expression levels might activate the mitochondrial apoptosis. The connection between Erk and Nrf2 translocation is too complicated. It was reported that ERK1/2 activity is required for stabilization of Nrf2 and subsequent activation of HO-1 transcription (Sun et al., 2015). In this study, the expression levels of *erk1/2* and *nrf2* were both downregulated. The decrease of *erk1/2* expression may be one of the reasons for the downregulation of *nrf2* expression. However, the connection between expression of *erk1/2* and *nrf2* in AC-induced developmental toxicity should be further studied. In the present study, we only detected mRNA expression without protein concentrations. This is subject to the limitations of zebrafish suitable antibodies. In the following research, we will verify the protein concentrations in rodent models, which are more credible.

Apoptosis plays an important role during embryogenesis and development. Mitochondrial outer membrane permeabilization and subsequent release of intermembrane space proteins to the cytosol are key events during apoptosis. The release of caspase-activating proteins during early apoptosis is regulated primarily by the Bcl-2 family of proteins (Gogvadze and Orrenius, 2006). Briefly, the activation of the Bcl-2 family led to the formation of a pore in the mitochondrial membrane. Mitochondrion then releases the proapoptotic proteins, such as cytochrome c, that result in the formation of the apoptosome. The apoptosome then activates initiation of caspase-9, which in turn leads to the activation of caspase-3 and thus induces apoptosis (Siddiqui et al., 2015). In the present study, AC treatment induced the activation of apoptotic pathways, resulting in the upregulation of a series of proapoptotic genes including *Bad*, *Bax*, *Cyto C*, *Casp-9*, and *Casp-3* and downregulation of antiapoptotic gene *Bcl-2*, and finally activated mitochondrial apoptosis in the zebrafish embryo. These results indicated that the oxidative stress and apoptosis played important roles in the developmental toxicity induced by AC in the zebrafish embryo. Although AC in the natural environment rarely reaches the high concentrations, our study gives us a better understanding of why raw *Aconitum* is strictly forbidden for pregnant women and is benefit to realize the mistake toxic reaction after taking overdosed AC-containing herbs.

## Conclusion

**FIGURE 8:**
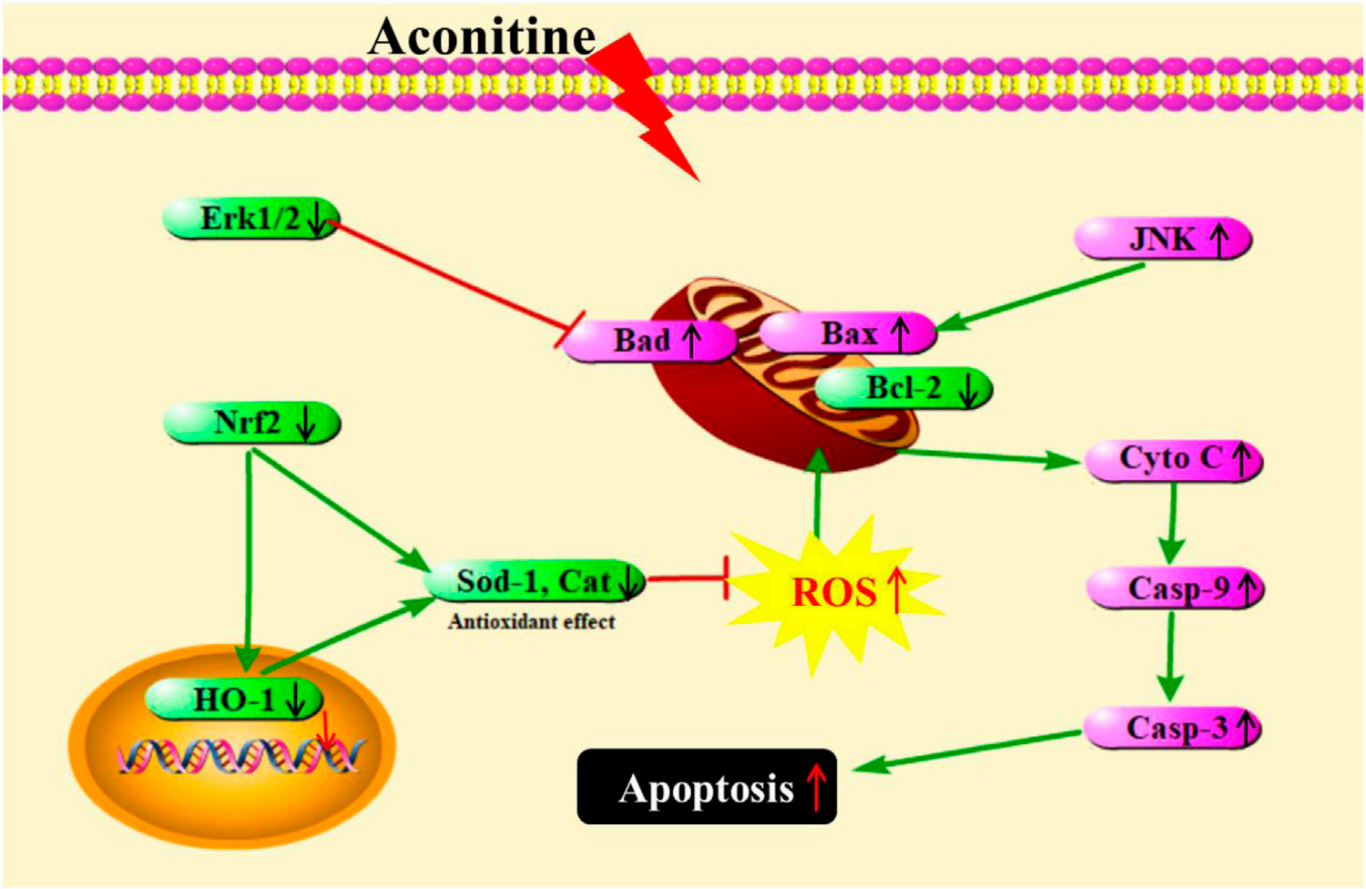
A schematic representation showing AC-induced developmental toxicity in zebrafish embryos through ROS-medicated mitochondrial apoptosis involving Nrf2/HO-1 and JNK/Erk pathways.

AC is a representative and extremely toxic compound in *Aconitum*. Although the cardiotoxicity and neurotoxicity have been recognized, the developmental toxicity of AC remains scarce. Our study showed that AC treatment caused reduced body length, curved body shape, high-malformation rate, pericardial edema, brain defects, yolk retention, and swim bladder defects. Further, AC also showed toxic effects on the developing heart, liver, and nervous system. Then, we discussed the potential molecular mechanisms for the first time. As summarized in Figure 8, AC induced developmental toxicity in zebrafish embryos through ROS-medicated mitochondrial apoptosis involving Nrf2/HO-1 and JNK/Erk pathways. These experimental findings may broaden our understanding of the AC toxic mechanism involved in AC therapeutics in humans. However, further comparative studies are necessary to elucidate the molecular mechanisms of AC in mammalian cell cultures.

## Data Availability

The raw data supporting the conclusion of this article will be made available by the authors, without undue reservation.

## References

[B1] Borcsa B. Widowitz U. Csupor D. Forgo P. Bauer R. Hohmann J. (2010). Semisynthesis and pharmacological investigation of lipo-alkaloids prepared from aconitine. Fitoterapia 82, 365–368. 10.1016/j.fitote.2010.11.001 21075183

[B2] Cassar S. Adatto I. Freeman J. L. Gamse J. T. Iturria I. Lawrence C. (2020). Use of zebrafish in drug discovery toxicology. Chem. Res. Toxicol. 33, 95–118. 10.1021/acs.chemrestox.9b00335 31625720 PMC7162671

[B3] Chan T. Y. K. (2009). Aconite poisoning. Clin. Toxicol. 47, 279–285. 10.1080/15563650902904407 19514874

[B4] Chan T. Y. K. (2016). AconitumAlkaloid poisoning because of contamination of herbs by aconite roots. Phytother. Res. 30, 3–8. 10.1002/ptr.5495 26481590

[B5] Cheng Y. Qiu F. Tashiro S.-i. Onodera S. Ikejima T. (2008). ERK and JNK mediate TNFα-induced p53 activation in apoptotic and autophagic L929 cell death. Biochem. Biophysical Res. Commun. 376, 483–488. 10.1016/j.bbrc.2008.09.018 18796294

[B6] d’Amora M. Giordani S. (2018). The utility of zebrafish as a model for screening developmental neurotoxicity. Front. Neurosci. 12, 976. 10.3389/fnins.2018.00976 30618594 PMC6305331

[B7] Deavall D. G. Martin E. A. Horner J. M. Roberts R. (2012). Drug-induced oxidative stress and toxicity. J. Toxicol. 2012, 645460. 10.1155/2012/645460 22919381 PMC3420138

[B8] Goessling W. Sadler K. C. (2015). Zebrafish: an important tool for liver disease research. Gastroenterology 149, 1361–1377. 10.1053/j.gastro.2015.08.034 26319012 PMC4762709

[B9] Gogvadze V. Orrenius S. (2006). Mitochondrial regulation of apoptotic cell death. Chemico-Biological Interactions 163, 4–14. 10.1016/j.cbi.2006.04.010 16730343

[B10] Ji B. L. Xia L. P. Zhou F. X. Mao G. Z. Xu L. X. (2016). Aconitine induces cell apoptosis in human pancreatic cancer via NF-κB signaling pathway. Eur. Rev. Med. Pharmacol. Sci. 20, 4955–4964. 27981537

[B11] Kamata H. Honda S.-i. Maeda S. Chang L. Hirata H. Karin M. (2005). Reactive oxygen species promote tnfα-induced death and sustained JNK activation by inhibiting MAP kinase phosphatases. Cell 120, 649–661. 10.1016/j.cell.2004.12.041 15766528

[B12] Li M. Xie X. Chen H. Xiong Q. Tong R. Peng C. (2020a). Aconitine induces cardiotoxicity through regulation of calcium signaling pathway in zebrafish embryos and in H9c2 cells. J. Appl. Toxicol. 40 (6), 780–793. 10.1002/jat.3943 31975431

[B13] Li Y. Ding H. Liu L. Song Y. Du X. Feng S. (2020b). Non-esterified fatty acid Induce dairy cow hepatocytes apoptosis via the mitochondria-mediated ROS-JNK/ERK signaling pathway. Front. Cell Dev. Biol. 8, 245. 10.3389/fcell.2020.00462 32411699 PMC7198733

[B14] Liu F. Han X. Li N. Liu K. Kang W. (2019). Aconitum alkaloids induce cardiotoxicity and apoptosis in embryonic zebrafish by influencing the expression of cardiovascular relative genes. Toxicol. Lett. 305, 10–18. 10.1016/j.toxlet.2019.01.002 30639578

[B15] Liu S. Li F. Li Y. Li W. Xu J. Du H. (2017). A review of traditional and current methods used to potentially reduce toxicity of Aconitum roots in Traditional Chinese Medicine. J. Ethnopharmacol. 207, 237–250. 10.1016/j.jep.2017.06.038 28666834

[B16] Loboda A. Damulewicz M. Pyza E. Jozkowicz A. Dulak J. (2016). Role of Nrf2/HO-1 system in development, oxidative stress response and diseases: an evolutionarily conserved mechanism. Cell. Mol. Life Sci. 73, 3221–3247. 10.1007/s00018-016-2223-0 27100828 PMC4967105

[B17] Ma Q. (2013). Role of nrf2 in oxidative stress and toxicity. Annu. Rev. Pharmacol. Toxicol. 53, 401–426. 10.1146/annurev-pharmtox-011112-140320 23294312 PMC4680839

[B18] Miller G. W. Chandrasekaran V. Yaghoobi B. Lein P. J. (2018). Opportunities and challenges for using the zebrafish to study neuronal connectivity as an endpoint of developmental neurotoxicity. Neurotoxicology 67, 102–111. 10.1016/j.neuro.2018.04.016 29704525 PMC6177215

[B19] Nyirimigabo E. Xu Y. Li Y. Wang Y. Agyemang K. Zhang Y. (2015). A review on phytochemistry, pharmacology and toxicology studies of Aconitum. J. Pharm. Pharmacol. 67, 1–19. 10.1111/jphp.12310 25244533

[B20] Orrenius S. Gogvadze V. Zhivotovsky B. (2007). Mitochondrial oxidative stress: implications for cell death. Annu. Rev. Pharmacol. Toxicol. 47, 143–183. 10.1146/annurev.pharmtox.47.120505.105122 17029566

[B21] Orrenius S. Zhivotovsky B. Nicotera P. (2003). Regulation of cell death: the calcium-apoptosis link. Nat. Rev. Mol. Cell. Biol. 4, 552–565. 10.1038/nrm1150 12838338

[B22] Peng F. Zhang N. Wang C. Wang X. Huang W. Peng C. (2019). Aconitine induces cardiomyocyte damage by mitigating BNIP3‐dependent mitophagy and the TNFα‐NLRP3 signalling axis. Cell Proliferat. 53, e12701. 10.1111/cpr.12701 PMC6985658 31657084

[B23] Siddiqui W. A. Ahad A. Ahsan H. (2015). The mystery of BCL2 family: Bcl-2 proteins and apoptosis: an update. Arch. Toxicol. 89, 289–317. 10.1007/s00204-014-1448-7 25618543

[B24] Singhuber J. Zhu M. Prinz S. Kopp B. (2009). Aconitum in Traditional Chinese Medicine-A valuable drug or an unpredictable risk? J. Ethnopharmacol. 126, 18–30. 10.1016/j.jep.2009.07.031 19651200

[B25] Sinha K. Das J. Pal P. B. Sil P. C. (2013). Oxidative stress: the mitochondria-dependent and mitochondria-independent pathways of apoptosis. Arch. Toxicol. 87, 1157–1180. 10.1007/s00204-013-1034-4 23543009

[B26] Song L. R. Hong X. Ding X. L. Zang Z. Y. (2001). Modern dictionary of Chinese materia medica (xiandai zhongyao dacidian). Beijing, China: People’s Medical Publishing House (Renmin Weisheng Chubanshe).

[B27] Song Z. Zhang Y. Zhang H. Rajendran R. S. Wang R. Hsiao C. D. (2020). Isoliquiritigenin triggers developmental toxicity and oxidative stress-mediated apoptosis in zebrafish embryos/larvae via Nrf2-HO1/JNK-ERK/mitochondrion pathway. Chemosphere 246, 12. 10.1016/j.chemosphere.2019.125727 31896010

[B28] Sun G.-b. Sun H. Meng X.-b. Hu J. Zhang Q. Liu B. (2014). Aconitine-induced Ca^2+^ overload causes arrhythmia and triggers apoptosis through p38 MAPK signaling pathway in rats. Toxicol. Appl. Pharmacol. 279, 8–22. 10.1016/j.taap.2014.05.005 24840785

[B29] Sun G. Y. Chen Z. Jasmer K. J. Chuang D. Y. Gu Z. Hannink M. (2015). Quercetin attenuates inflammatory responses in BV-2 microglial cells: role of MAPKs on the Nrf2 pathway and induction of heme oxygenase-1. PLoS One 10, e0141509. 10.1371/journal.pone.0141509 26505893 PMC4624710

[B30] Sun Z. Huang Z. Zhang D. D. (2009). Phosphorylation of Nrf2 at multiple sites by MAP kinases has a limited contribution in modulating the Nrf2-dependent antioxidant response. PLoS One 4, e6588. 10.1371/journal.pone.0006588 19668370 PMC2719090

[B31] Tang L. Gong Y. Lv C. Ye L. Liu L. Liu Z. (2012). Pharmacokinetics of aconitine as the targeted marker of Fuzi (Aconitum carmichaeli) following single and multiple oral administrations of Fuzi extracts in rat by UPLC/MS/MS. J. Ethnopharmacol. 141, 736–741. 10.1016/j.jep.2011.08.070 21924342

[B32] Thongsom S. Suginta W. Lee K. J. Choe H. Talabnin C. (2017). Piperlongumine induces G2/M phase arrest and apoptosis in cholangiocarcinoma cells through the ROS-JNK-ERK signaling pathway. Apoptosis 22, 1473–1484. 10.1007/s10495-017-1422-y 28913568

[B33] Wada K. Nihira M. Hayakawa H. Tomita Y. Hayashida M. Ohno Y. (2004). Effects of long-term administrations of aconitine on electrocardiogram and tissue concentrations of aconitine and its metabolites in mice. Forensic Sci. Int. 148, 21–29. 10.1016/j.forsciint.2004.04.016 15607586

[B34] Wang M.-Y. Liang J.-W. Olounfeh K. Sun Q. Zhao N. Meng F.-H. (2018). A comprehensive in silico method to study the QSTR of the aconitine alkaloids for designing novel drugs. Molecules 23, 2385. 10.3390/molecules23092385 PMC6225272 30231506

[B35] Wang M. Shi Y. Yao L. Li Q. Wang Y. Fu D. (2020). Potential molecular mechanisms and drugs for aconitine-induced cardiotoxicity in zebrafish through RNA sequencing and bioinformatics analysis. Med. Sci. Monit. 26, e924092. 10.12659/msm.924092 32598336 PMC7341694

[B36] Waza A. A. Hamid Z. Ali S. Bhat S. A. Bhat M. A. (2018). A review on heme oxygenase-1 induction: is it a necessary evil. Inflamm. Res. 67, 579–588. 10.1007/s00011-018-1151-x 29693710

[B37] Wright S. N. (2002). Comparison of aconitine‐modified human heart (hH1) and rat skeletal (μ1) muscle Na + channels: an important role for external Na + ions. J. Physiol. 538, 759–771. 10.1113/jphysiol.2001.012915 11826163 PMC2290112

[B38] Xia Q. Wei L. Zhang Y. Kong H. Shi Y. Wang X. (2018). Psoralen induces developmental toxicity in zebrafish embryos/larvae through oxidative stress, apoptosis, and energy metabolism disorder. Front. Pharmacol. 9, 1457. 10.3389/fphar.2018.01457 30618751 PMC6305401

[B39] Xiao K. Wang L. Liu Y. Peng C. Yan G. Zhang J. (2007). Study of aconitine toxicity in rat embryos *in vitro* . Birth Defect Res. B 80, 208–212. 10.1002/bdrb.20116 17570135

[B40] Ye Q. Liu H. Fang C. Liu Y. Liu X. Liu J. (2019). Cardiotoxicity evaluation and comparison of diterpene alkaloids on zebrafish. Drug Chem. Toxicol. 21, 1–8. 10.1080/01480545.2019.1586916 30895830

[B41] Zhen Y.-S. Wu Q. Xiao C.-L. Chang N.-N. Wang X. Lei L. (2012). Overlapping cardiac programs in heart development and regeneration. J. Genet. Genomics 39, 443–449. 10.1016/j.jgg.2012.07.005 23021544

[B42] Zhou G. Tang L. Zhou X. Wang T. Kou Z. Wang Z. (2015). A review on phytochemistry and pharmacological activities of the processed lateral root of Aconitum carmichaelii Debeaux. J. Ethnopharmacol. 160, 173–193. 10.1016/j.jep.2014.11.043 25479152

